# Combined orcein and martius scarlet blue (OMSB) staining for qualitative and quantitative analyses of atherosclerotic plaques in brachiocephalic arteries in apoE/LDLR^−/−^ mice

**DOI:** 10.1007/s00418-017-1538-8

**Published:** 2017-02-06

**Authors:** Mariusz Gajda, Agnieszka Jasztal, Tomasz Banasik, Ewa Jasek-Gajda, Stefan Chlopicki

**Affiliations:** 1 Department of Histology, Jagiellonian University Medical College, Kopernika 7, 31-034 Kraków, Poland; 2 Jagiellonian Centre for Experimental Therapeutics (JCET), Jagiellonian University, Bobrzyńskiego 14, 30-348 Kraków, Poland; 3 Chair of Pharmacology, Jagiellonian University Medical College, 31-531 Kraków, Poland

**Keywords:** Atherosclerosis, Elastic fibers, Polychrome staining, Orcein, Martius scarlet blue, ApoE/LDLR^−/−^ mice

## Abstract

Numerous cellular and extracellular components should be analyzed in sections of atherosclerotic plaques to assess atherosclerosis progression and vulnerability. Here, we combined orcein (O) staining for elastic fibers and martius scarlet blue (MSB) polychrome to visualize various morphological contents of plaque in brachiocephalic arteries (BCA) of apoE/LDLR^−/−^ mice. Elastic fibers (including broken elastic laminae and ‘buried’ fibrous caps) were stained purple and they could be easily distinguished from collagen fibers (blue). Orcein allowed clear identification of even the finest elastic fibers. Erythrocytes were stained yellow and they could easily be discerned from mature fibrin (red). Old fibrin tends to acquire blue color. The method of OMSB staining is simple, takes less than 1 h to perform and can be adapted to automatic stainers. Most importantly, the color separation is good enough to allow digital automatic segmentation of specific components in tissue section and quantitative analysis of the plaque constituents. OMSB was used to compare atherosclerotic plaques in proximal and distal regions of BCA in apoE/LDLR^−/−^ mice. In conclusion, OMSB staining represents a novel staining that could be routinely used for qualitative and quantitative microscopic assessments of formaldehyde-fixed and paraffin-embedded sections of arteries with atherosclerotic lesions.

## Introduction

Gene targeted apoE^−/−^ or apoE/LDLR^−/−^ mice that spontaneously develop atherosclerotic plaques represent suitable animal models for studies on dietary and pharmacological treatments of atherosclerosis (for review see: Getz and Reardon 2015). At the present time, measuring the area of tissue stained with oil red O in frozen sections is the most common approach to quantify atherosclerotic lesions in apoE/LDLR^−/−^ mice. Unfortunately, this technique cannot provide the necessary histological detail that determines plaque vulnerability. On the other hand, comprehensive histological assessment of arteries with atherosclerotic plaques represents the most reliable method of qualitative and quantitative evaluation of the atherosclerosis. Such an assessment allows to analyze various cellular and extracellular components of the atherosclerotic plaque: smooth muscle cells, macrophages (with foam cells), connective tissue fibers (elastic and collagen), lipids, mineral concretions, fibrin deposits, and intraplaque erythrocytes. Altered elastic fibers (including ‘buried’ fibrous caps and broken elastic laminae) and intraplaque hemorrhages observed as deposits of erythrocytes and fibrin may result from plaque rupture, and they are considered by some authors as determinants of unstable plaque (Jackson et al. 2007; Sluimer et al. 2015). All the above components can be demonstrated on separate tissue sections using histological, histochemical, and immunohistochemical staining methods. However, the number of sections available for histological processing of brachiocephalic artery (BCA) or other affected arteries is often limited. Thus, polychrome stainings allowing simultaneous demonstration of various tissue components may be preferred for morphological analysis of the plaques, in particular if such methods are characterized by distinct color separation that allows clear differentiation between specific tissue components. Martius yellow–crystal scarlet–methyl blue (MSB, Lendrum et al. 1962) is a polychrome stain originally designed for the detection of fibrin. This method also gives specific and distinct staining pattern of collagen fibers and erythrocytes. We supplemented MSB staining with orcein to allow additional detection of elastic fibers.

In this study, we describe combining orcein with MSB (OMSB) to provide versatile polychromatic staining, suitable for qualitative and quantitative analyses of formalin-fixed and paraffin-embedded sections of atherosclerotic arteries in mice. To demonstrate advantage of this method, OMSB stained BCAs were automatically segmented to compare size and histological composition of atherosclerotic plaques in proximal and distal regions of the vessel.

## Materials and methods

### Animals and tissue samples

ApoE/LDLR^−/−^ mice were originally obtained from Jackson Laboratory (Sacramento, CA, USA; for reference, see: Ishibashi et al. 1994) and bred in house. Animals were kept in colony cages, in a temperature-controlled environment (22–25 °C) with a 12h:12h light/dark cycle. They had free access to food (cholesterol-free, pelleted diet; Sniff M-Z Spezialdiäten GmbH, Soest, Germany) and water.

All procedures involving animals were conducted according to the Guidelines for Animal Care and Treatment of the European Union and they were approved by the First Local Ethics Commission for Animal Experiments in Kraków (declaration No. 51/2009).

Six-month-old (established atherosclerosis) apoE/LDLR^−/−^ mice were used in this study. The animals were euthanized intraperitoneally with 100 mg/kg ketamine/10 mg/kg xylazine. Brachiocephalic arteries were dissected out, fixed in 4% buffered paraformaldehyde (pH = 7.4) for at least 48 h, and then routinely processed and embedded in paraffin (tissue processor TP1020; Leica, Wetzlar, Germany). Cross sections were cut every 5 μm along the artery (starting from the proximal end) and mounted serially on silanized slides. Sections were stained and mounted automatically using Leica ST5010 Autostainer XL combined with Leica CV5030 Glass Coverslipper.

### Staining solutions

1. Orcein solution:

Dissolve 1 g orcein (Natural red 28, synthetic; Sigma-Aldrich, #O7380) in 80 ml 96% ethyl alcohol, 19 ml water, and 1 ml HCl.

2. Martius yellow solution:

Dissolve 0.5 g martius yellow (Acid yellow 24, C.I. 10315; Sigma-Aldrich, #287814) in 100 ml 96% alcohol and then add 2 g of phosphotungstic acid.

3. Crystal scarlet solution:

Dissolve 1 g brilliant crystal scarlet (Acid red 44, C.I. 16250; Sigma-Aldrich, #C0644) in 97.5 ml distilled water with 2.5 ml glacial acetic acid.

4. Phosphotungstic acid solution:

Dissolve 1 g phosphotungstic acid in 100 ml distilled water.

5. Methyl blue solution:

Dissolve 0.5 g methyl blue (Acid blue 94, C.I. 42780; Sigma-Aldrich, #95290) in 99 ml distilled water with 1 ml glacial acetic acid.

6. Acidic alcohol:

Mix 1 ml of hydrochloric acid with 99 ml of 96% ethyl alcohol.

7. Acetic acid water:

Mix 0.5 ml glacial acetic acid with 99.5 ml distilled water.

### Staining protocol

Deparaffinize sections through xylene and ethanol, and rehydrate to water. Postfix sections in Bouin’s fixative at 56 °C for 1 h (procedure used for non-mercuric chloride fixed sections, also applied by Howat and Wilson 2014), then rinse for 10 min in tap water to remove traces of picric acid. In contrast to the original formula (Lendrum et al. 1962), we omitted step of nuclear staining to improve the recognition of elastic fibers for automated segmentation (also applied by Howat and Wilson 2014). Stain with orcein for 20 min at 56 °C. Differentiate in acidic alcohol for 2 min and then transfer to 96% ethanol for 30 s. Stain with martius yellow for 3 min and rinse in distilled water (2 × 2 min). Stain with crystal scarlet for 10 min. Differentiate with phosphotungstic acid 2 min and transfer to distilled water (for about 10 s). Stain with methyl blue 5 min. Rinse in acetic acid water 3 min, dehydrate through ethanol and carbol-xylene, clear in xylene, and mount in DPX or other resin.

Specificity of collagen and fibrin detection was confirmed using other methods. Staining of fibrin was compared with phosphotungstic acid hematoxylin (PTAH) and of collagen with picrosirius red.

### Microscopy and recordings

The sections were examined and digital images of stained arteries were recorded using Olympus dotSlide system (Olympus, Tokyo, Japan).

### Image analysis and automatic segmentation

A simple protocol for automatic segmentation of tissue components in affected vessels was developed in Acapella Language provided by Columbus 2.4 package (Perkin Elmer, Waltham, MA). In this algorithm, first, the image mask was calculated by thresholding the color of elastic fibers (purple). The mask contained not only elastic fibers but also cell nuclei, fibrin, and foam cells. Elastic laminae in vessel media were recognized by their geometrical properties and texture. Elastic laminae mask was then processed using morphological procedures to finally obtain a mask for the vessel media that was defined as area between the innermost and outermost elastic lamina. The inner elastic lamina outlines internal vessel area occupied by plaque and lumen. Next step was to divide this area between the lumen and the plaque. The vessel lumen is clearly seen on original images as a white area, but it cannot be discerned from the lipid areas which are also white. The presence of erythrocytes (stained yellow) allowed to classify the given areas as vessel lumen. White areas with erythrocytes were masked as lumen and lipid areas were those containing no red blood cells. Plaque area was calculated by subtraction: internal vessel area minus lumen area. The last step was to mask collagen inside the plaque by blue color thresholding.

Segmentation algorithm allowed to distinguish vessel media, lumen, plaque area, and areas of the plaque occupied by collagen and lipids. The results were presented as areas of identified components in μm^2^. Further morphological parameters were calculated: total vessel area (media + lumen + plaque), relative lumen area (lumen area/total vessel area × 100%), relative plaque area (plaque area/total vessel area × 100%), relative collagen area (collagen area/plaque area × 100%), and relative lipid area (lipid area/plaque area × 100%). Totally, 150 sections from proximal and 150 sections from distal segments of BCA were analyzed applying above formula.

The color images showing results of segmentation overlying original images were created to visually inspect adequacy of automatic segmentation. To check the accuracy of automatic procedure, we performed free-hand drawing segmentation for 30 sections from proximal and 30 sections from distal regions of BCA. For the manual segmentation, we included following parameters: vessel media, vessel lumen, plaque area, and area occupied by lipids. Since collagen is dispersed in the plaque, the manual measurement of its area cannot be performed by this approach and the accuracy of collagen segmentation was assessed by comparison of images presenting results of automatic segmentation with original images by three independent observers.

Automatic and manual segmentations were used to compare morphological parameters in sections from proximal and distal segments of BCA. Mann–Whitney *U* test was applied to confirm differences (Prism 5.0, GraphPad, La Jolla, CA). *p* value less than 0.05 was considered statistically significant. Data were presented in the form of Tukey’s ‘box-and-whisker’ plots.

## Results

**Fig. 1:**
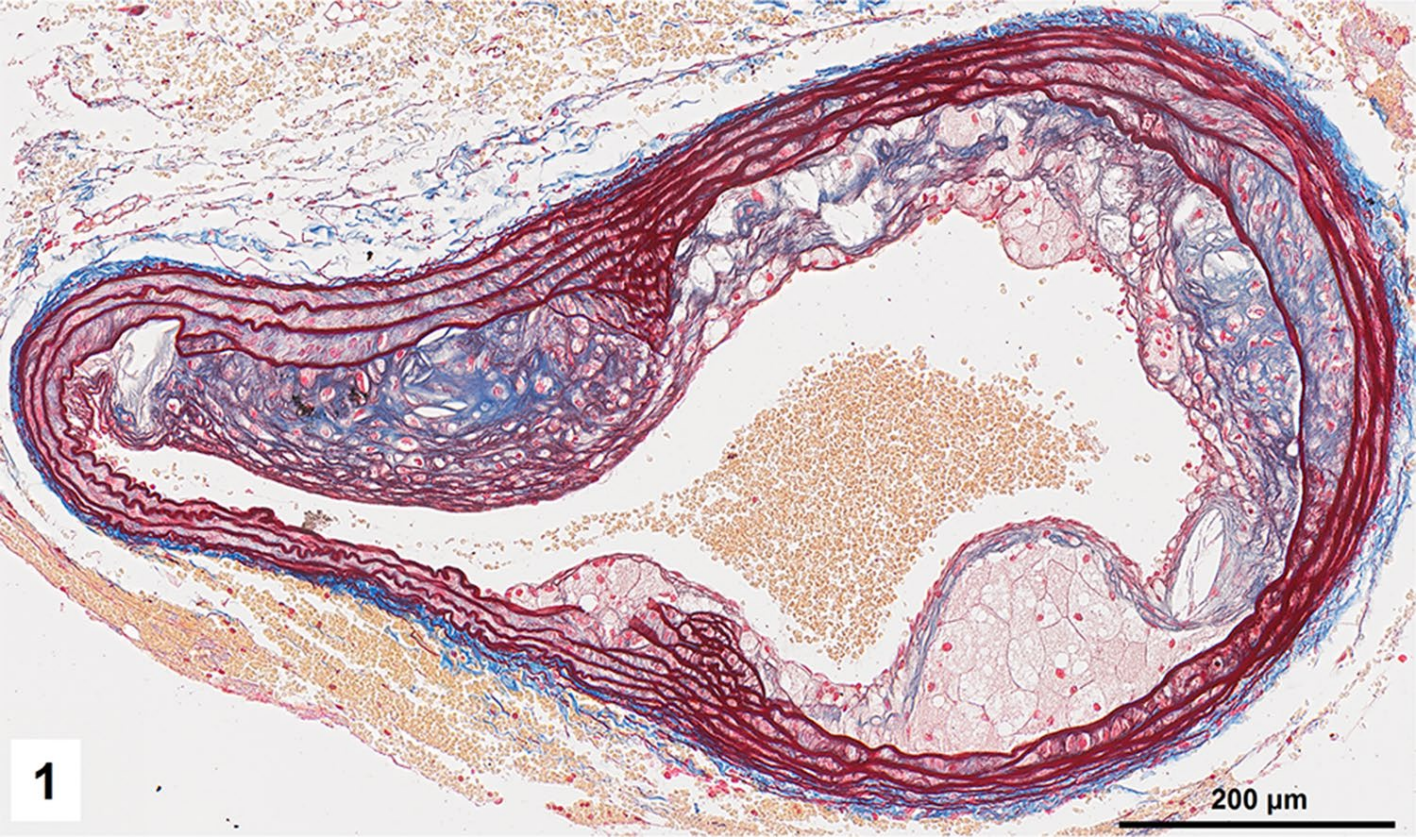
Overview of brachiocephalic artery of apoE/LDLR^−/−^ mouse presenting advanced atherosclerotic plaques

**Fig. 2:**
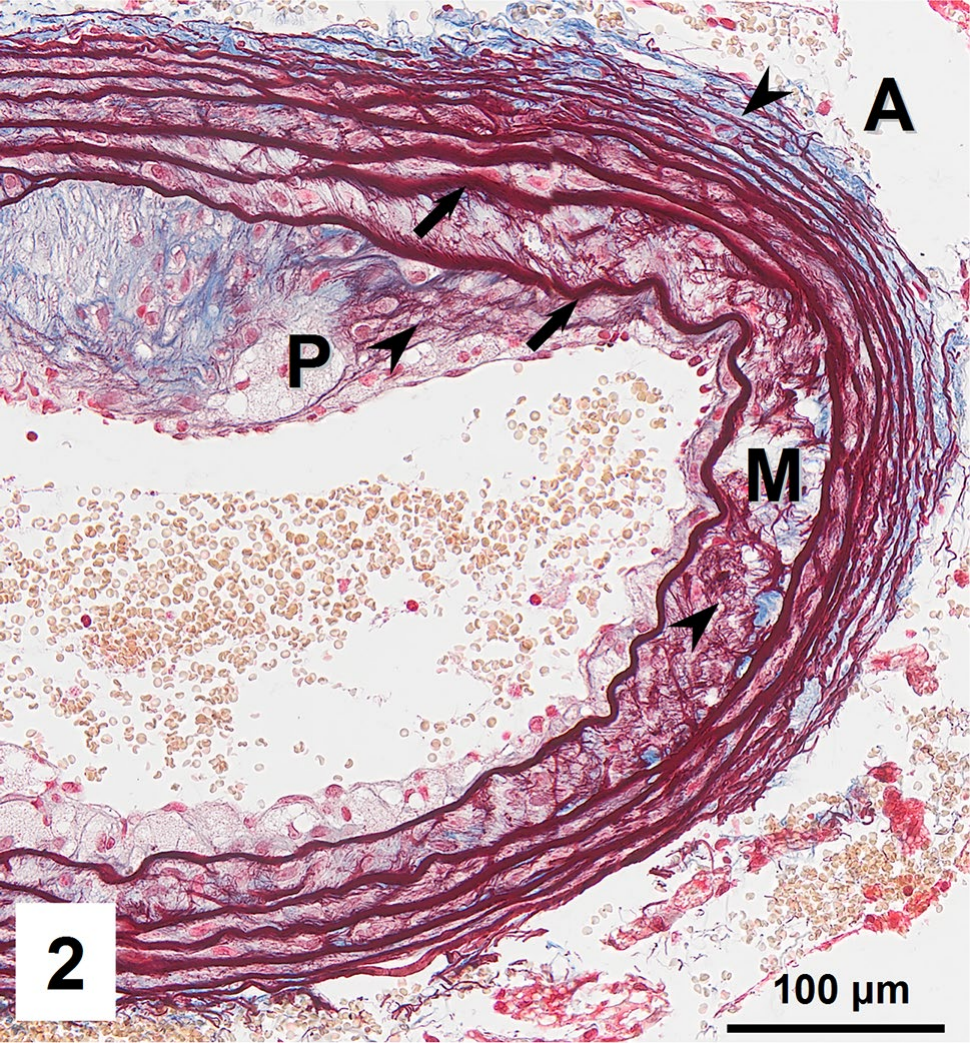
Elastic laminae (*arrows*) and fine elastic fibers (*arrowheads*) located in plaque (P), media (M), and adventitia (A) of the vessel

**Fig. 3:**
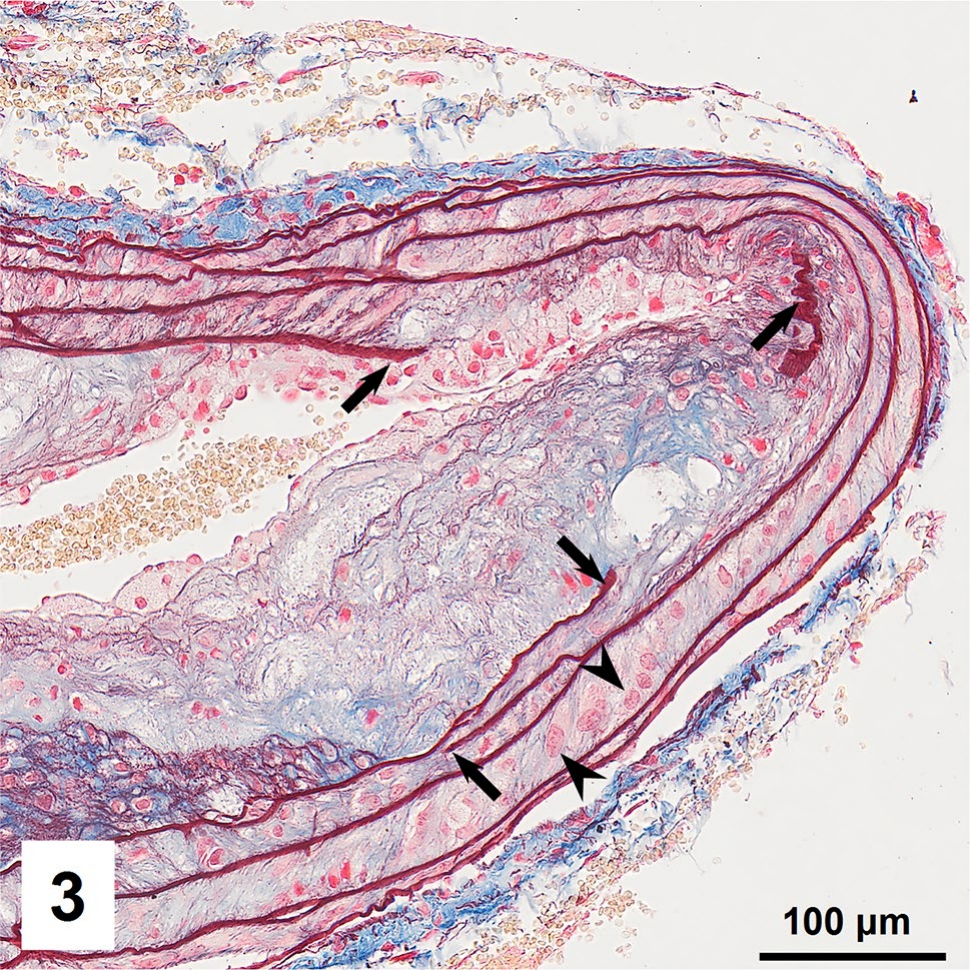
Broken elastic laminae (*arrows*) in vascular media. Vascular smooth muscle cells located between elastic laminae (*arrowheads*)

**Fig. 4:**
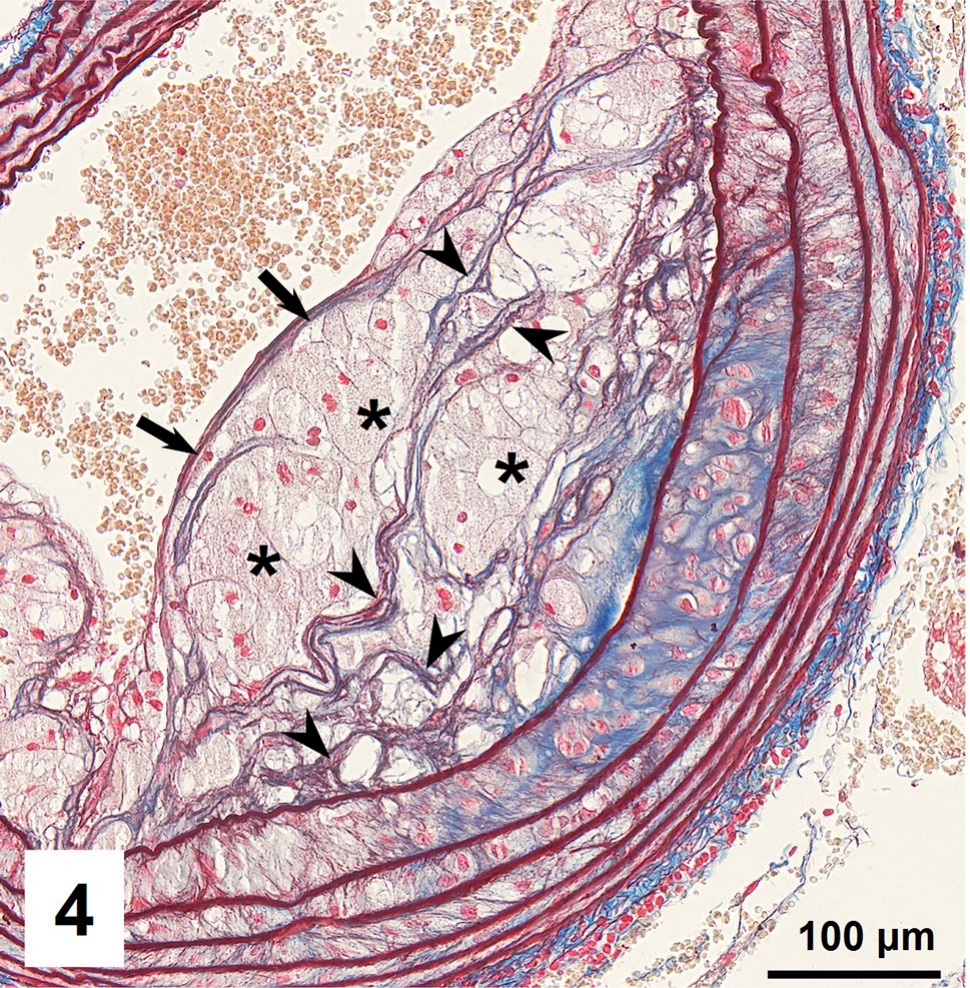
Fibrous caps (*arrows*) on the surface and ‘buried’ fibrous caps (*arrowheads*) within plaque rich in foam cells (*asterisks*)

**Fig. 5:**
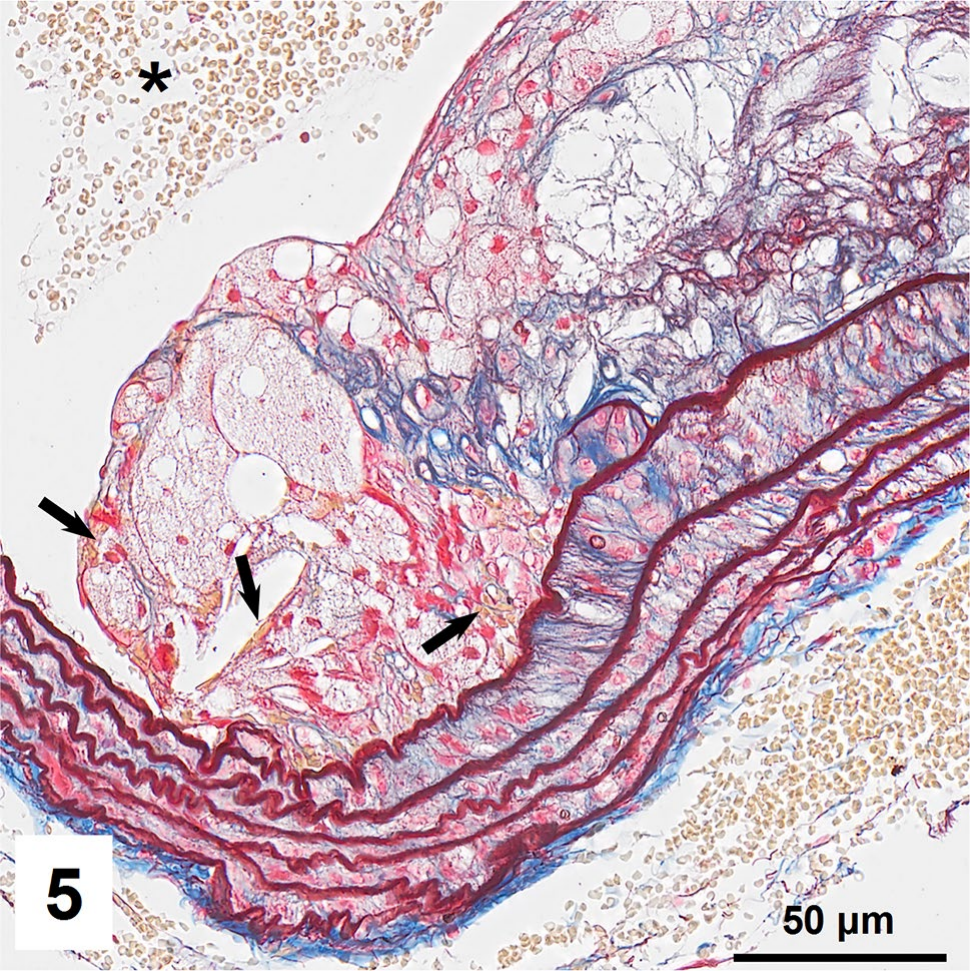
Erythrocytes present in the lumen of brachiocephalic artery (*asterisk*) and entrapped in plaque (*arrows*) within plaques rich in foam cells

**Fig. 6:**
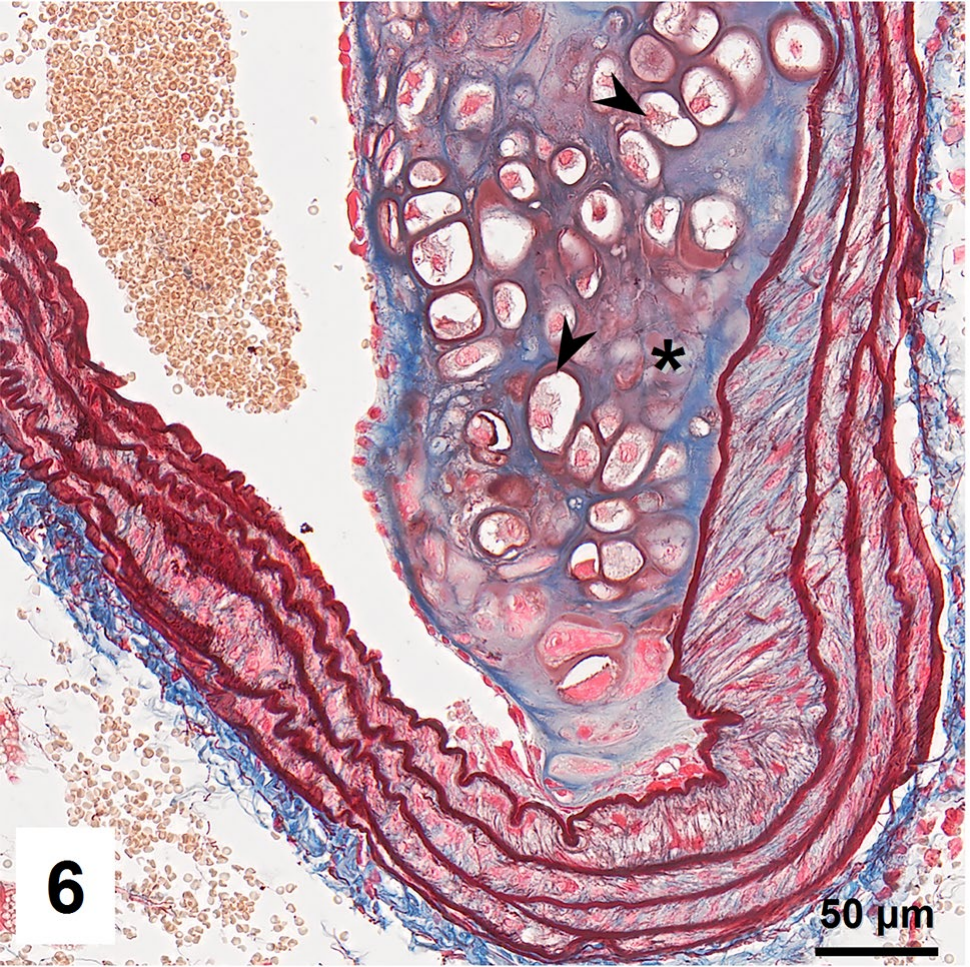
Plaque occupied by chondrocyte-like cells (*arrowheads*) and collagen rich matrix (*asterisk*; pseudochondroplasia)

**Fig. 7:**
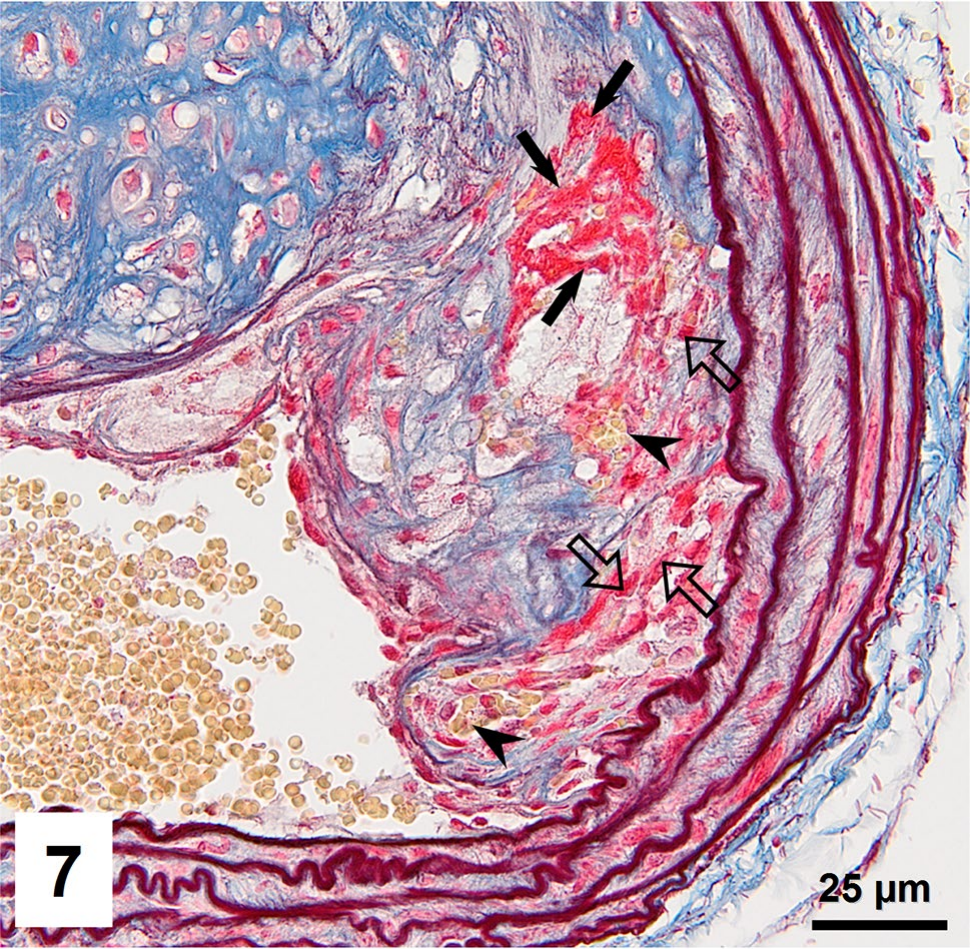
Mature fibrin (*arrow*) located in fatty streak lesion. Numerous intraplaque erythrocytes (*arrowheads*) are also visible. Cell nuclei are marked with *block arrows*

**Fig. 8:**
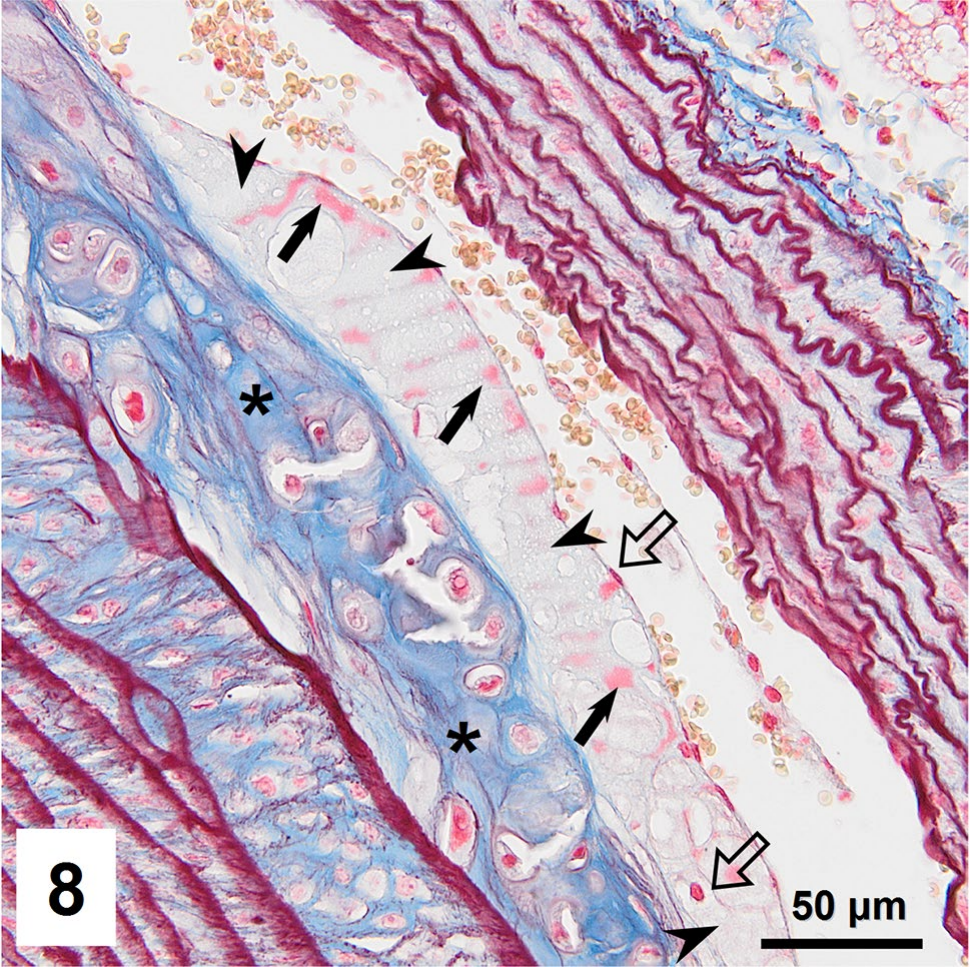
Subendothelial accumulation of mature (*red, arrows*) and old fibrin (*slightly bluish, arrowheads*). Pseudochondroplasia present in deeper region of neointima (*asterisks*). Cell nuclei are marked with *block arrows*

Combined orcein and MSB staining method (OMSB) discriminated selected components of atherosclerotic plaques in apoE/LDLR^−/−^ mice. Using this method, elastic fibers and elastic laminae were stained purple/grey and they could be easily distinguished from collagen fibers colored blue (Fig. 1). Orcein addition to the MSB staining allowed clear identification of even the finest elastic fibers (Fig. 2) and the staining was not masked or abolished by other dye solutions. OMSB staining demonstrated broken elastic laminae in media (Fig. 3), as well as elastic fibers located close to the vascular lumen (fibrous caps, Fig. 4) and within the plaques (so called ‘buried’ fibrous caps, Fig. 4). Erythrocytes located in the lumen of the vessel and those embedded in plaques were stained yellow (Figs. 5, 7). Smooth muscle cells were stained slightly red or pink (Fig. 3). Characteristic regions occupied by chondrocyte-like cells and cartilage matrix (pseudochondroplasia) were seen in the plaque areas (Figs. 6, 8). Extracellular matrix of pseudochondroplasia was stained blue due to the presence of collagen and it was easily discernible from the adjacent elastic fibers and muscle cells. Mature fibrin deposits were clearly seen in advanced lesions (Figs. 7, 8). They most frequently appeared intense red (Fig. 7) and they could be distinguished from erythrocytes stained yellow (Fig. 7) and old fibrin colored slightly blue (Fig. 8). Due to omitting nuclear staining, the cell nuclei were stained red. Since they present regular shape and characteristic texture of chromatin, they can be discerned from homogenously and more intensely stained mature fibrin by an experienced observer (Figs. 7, 8).

**Fig. 9:**
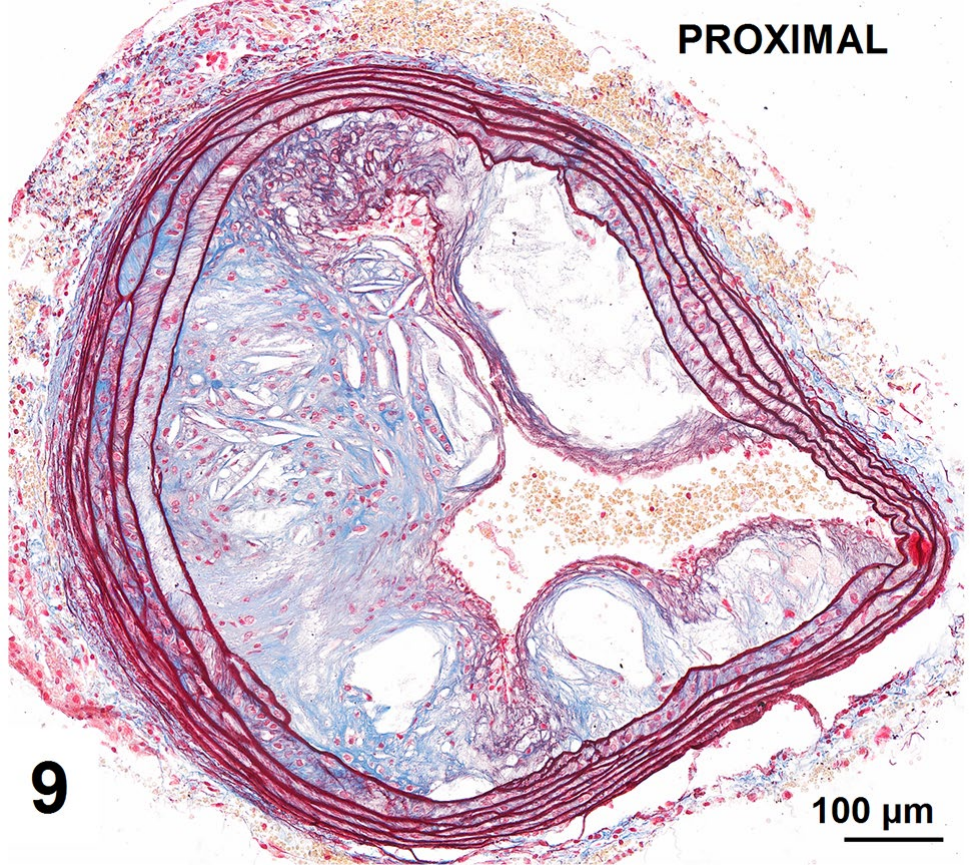
Advanced atherosclerotic plaques in proximal segment of brachiocephalic artery presenting mostly collagen deposits, pseudochondroplasia, fatty streaks, and no foam cells

**Fig. 10:**
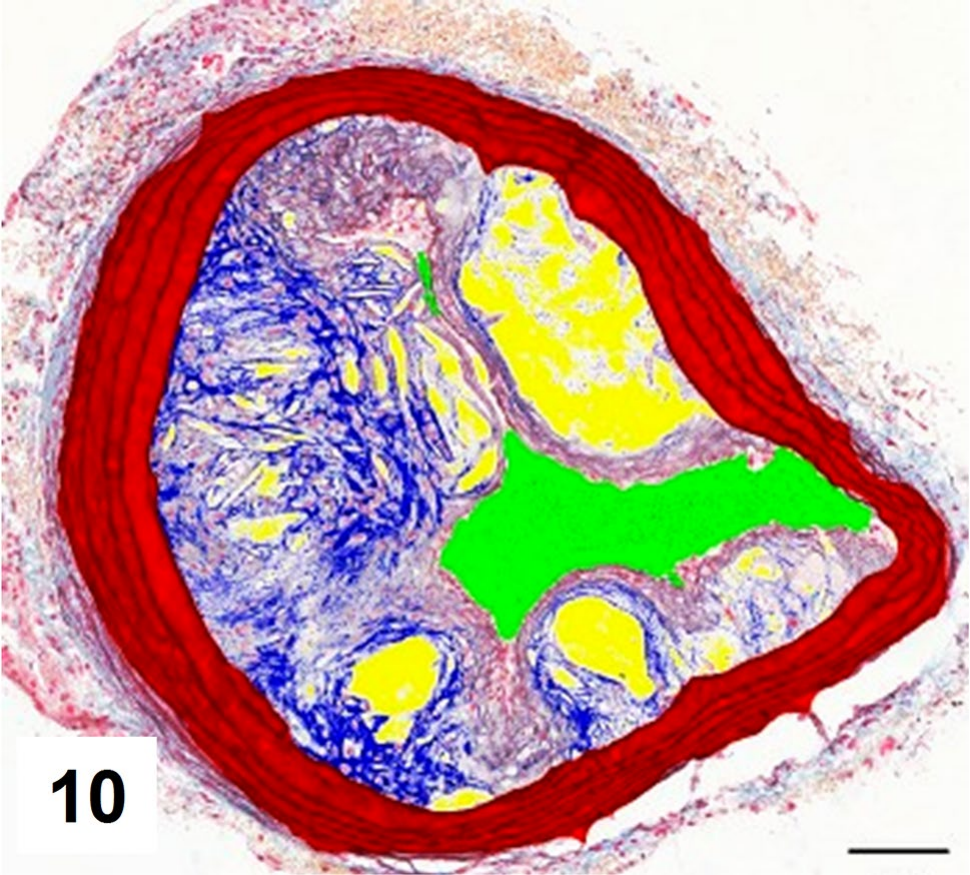
Results of automatic segmentation and pseudocoloring of the image from Fig. 9. Specific tissue areas overlying original image showing vessel media (*red* 161,749 μm^2^/33,58%), lumen (*green* 36,900 μm^2^/7,66%), plaque (calculated, 283,017 μm^2^/58,75%), collagen (*blue* 41,446 μm^2^/14,64%), and lipids (*yellow* 37,317 μm^2^/13,18%)

Automatic segmentation allowed clear detection of vessel media (pseudocolored red), lumen (pseudocolored green), and plaque components: collagen (pseudocolored blue) and lipids (pseudocolored yellow) (Figs. 9, 10, 12, 13). The recognized areas were then automatically quantified and results were presented as absolute areas of labeled components (μm^2^) as well as ratios of defined areas (%; data presented in legends to Figs. 10, 13). Total vessel area, vessel media, lumen, plaque, collagen, and lipid areas were higher in distal segment of BCA (Fig. 15a–f). Relative parameters showed statistical significance only for plaque ratio with higher area of the vessel occupied by atherosclerotic plaques in distal segment of BCA (Fig. 15h). 

**Fig. 11:**
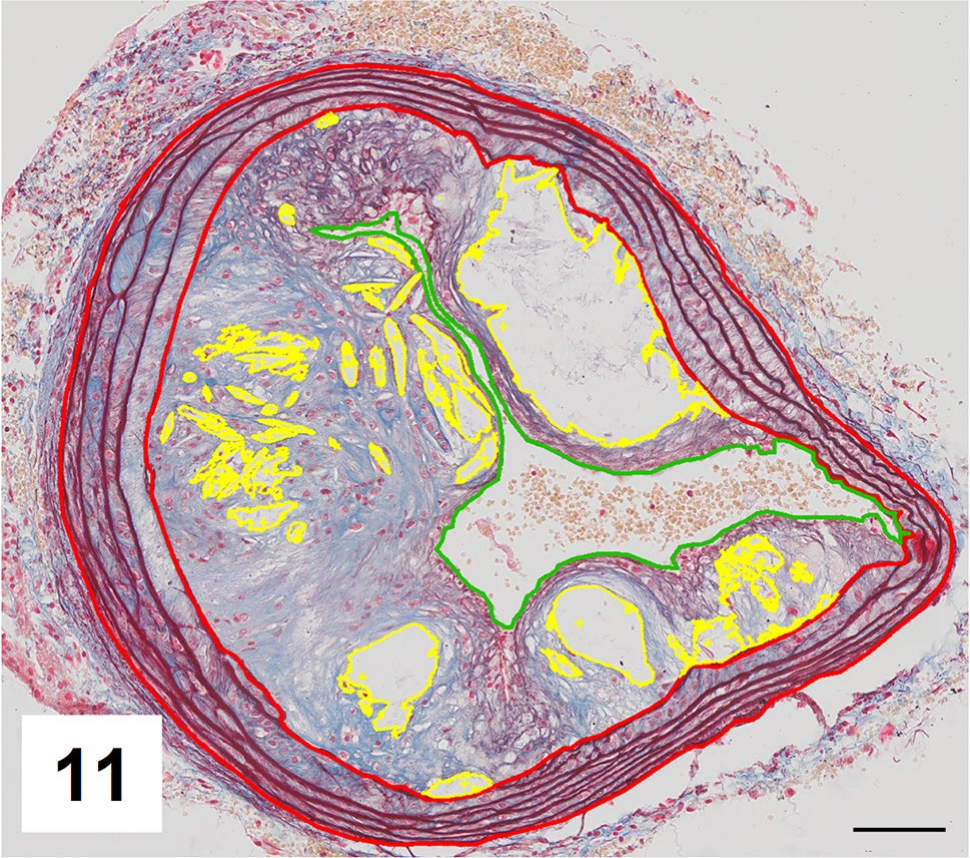
Results of manual segmentation of the image from Fig. 9. Specific tissue areas marked on original image showing vessel media (*red*, 155,723 μm^2^/32,46%), lumen of the vessel (*green*, 38,097 μm^2^/7,94%), plaque (calculated, 285,795 μm^2^/59,58%),  lipids (*yellow*, 39,553 μm^2^/13,83%)

The results of automatic segmentation were found to be reproducible and comparable to the free-hand drawing segmentation (Figs. 11, 14 and data presented in legends). Observed differences were ±5% for vessel media, vessel lumen, and plaque area, and <7% for lipids (automatic vs. manual, p > 0.5, U test). The accuracy of collagen segmentation was confirmed by three independent observers. They agreed that areas marked by automatic processing well match areas stained with methyl blue.

**Fig. 12:**
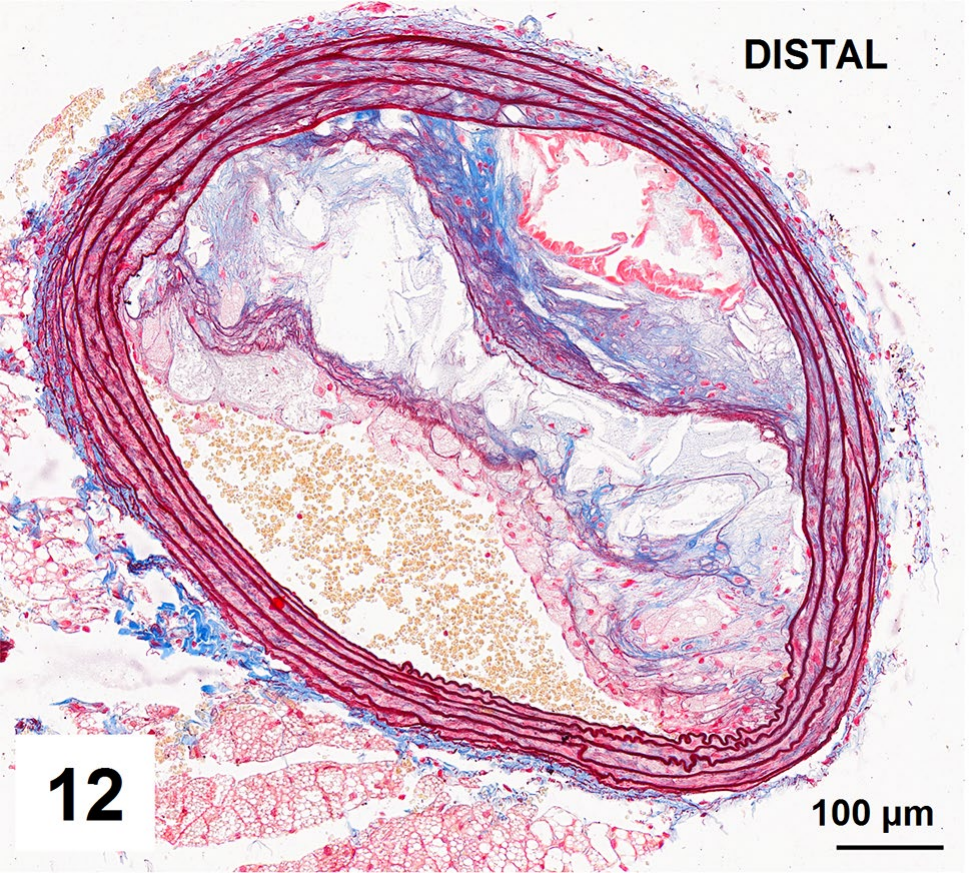
Advanced atherosclerotic plaques in distal segment of brachiocephalic artery presenting mostly fatty streaks, foam cells, fibrin deposits and in less extent collagen deposits and pseudochondroplasia

**Fig. 13:**
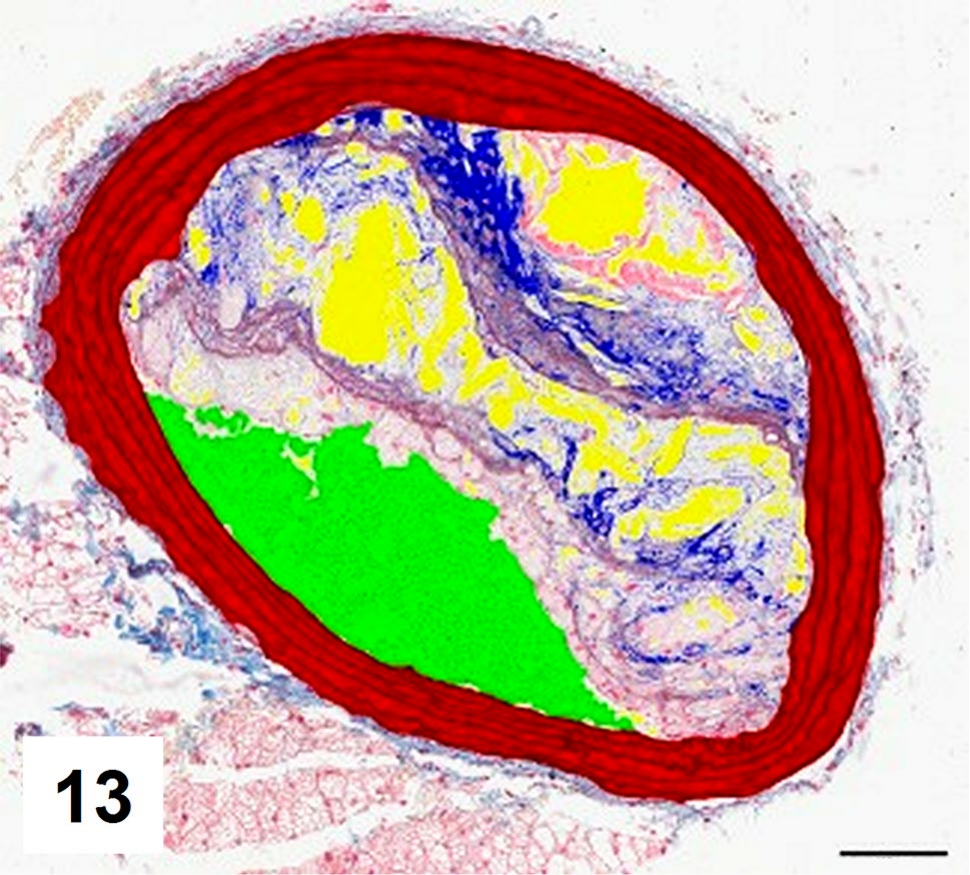
Results of automatic segmentation and pseudocoloring of the image from Fig. 12. Specific tissue areas overlying original image showing vessel media (*red*, 130,864 μm^2^/31,84%), lumen (*green*, 55,146 μm^2^/13,42%), plaque (calculated, 224,918 μm^2^/54,73%), collagen (*blue*, 18,018 μm^2^/8,01 %), lipids (*yellow*, 33,820 μm^2^/15,03%)

**Fig. 14:**
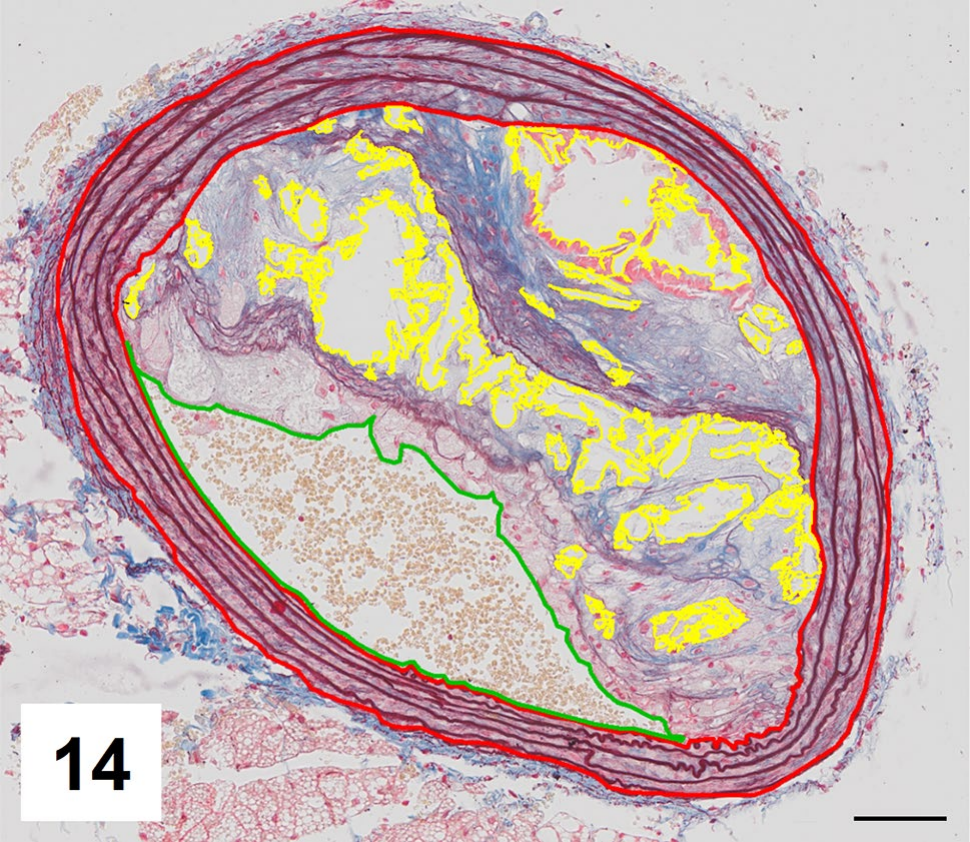
Results of manual segmentation of the image from Fig. 12. Specific tissue areas marked on original image showing vessel media (*red*, 128,424 μm^2^/31,19%), lumen of the vessel (*green*, 57,445 μm^2^/13,95%), plaque (calculated, 225,778 μm^2^/54,84%),  lipids (*yellow*, 34,781 μm^2^/15,4%)

**Fig. 15:**
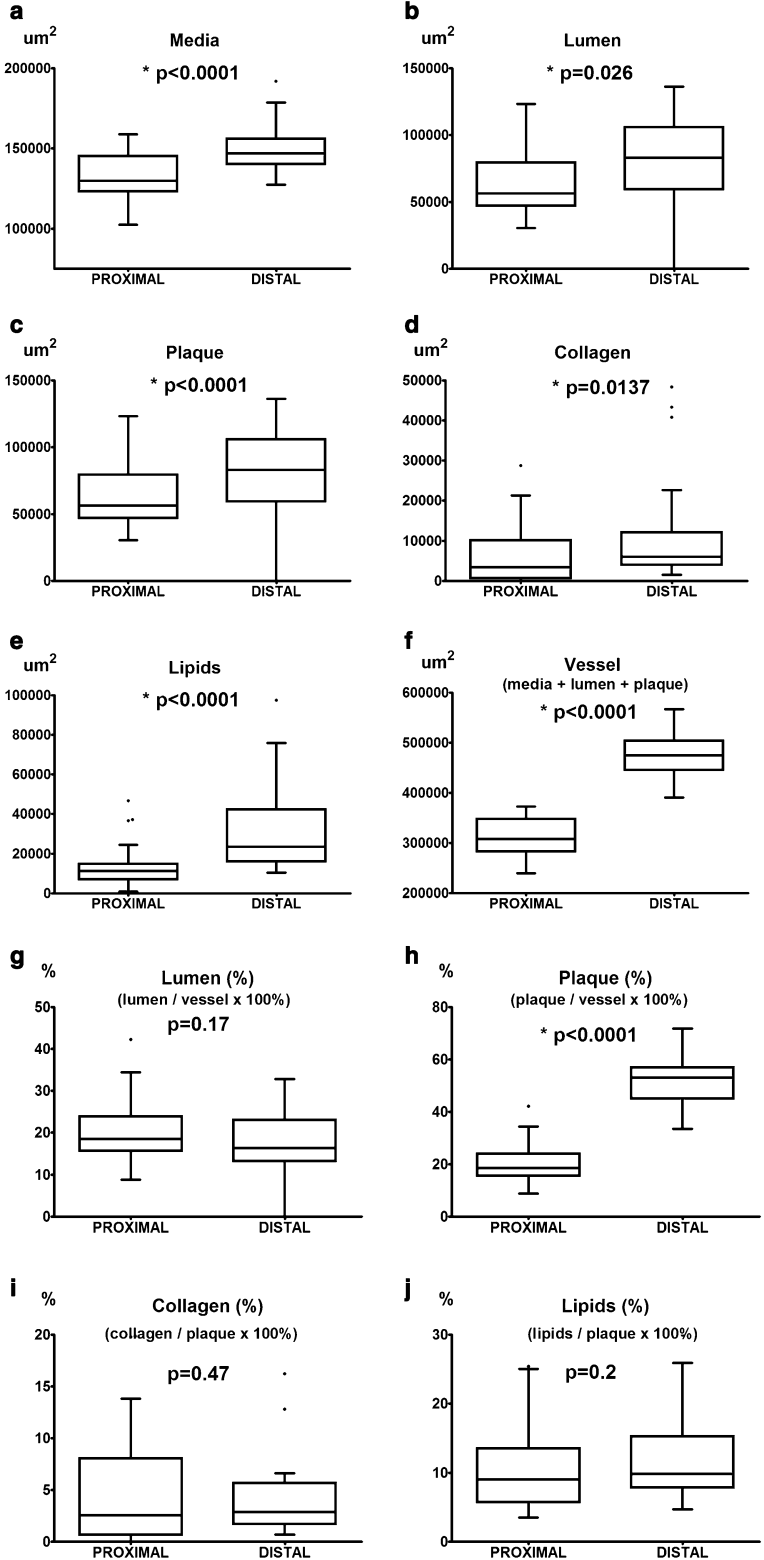
Comparison of morphological parameters between proximal and distal segments of brachiocephalic artery: areas of vessel media (**a**), lumen (**b**), plaque (**c**), collagen (**d**), lipids (**e**), total vessel area (**f**) and relative areas of lumen (**g**), plaque (**h**), collagen (**i**) and lipids (**j**)

## Discussion

This study reports a simple and reliable method—OMSB staining—designed for simultaneous detection of key tissue components in atherosclerotic plaques and thus it is suitable for both qualitative and quantitative analyses.

Combined orcein and MSB staining identifies and clearly differentiates collagen, elastic fibers, fatty streaks, fibrin, foam cells, and intraplaque erythrocytes. OMSB visualizes elastic fibers, as well as it provides selective staining of intraplaque erythrocytes and fibrin, all being markers of vulnerable plaques. To our knowledge, none of the original polychrome methods for the connective tissue described so far have these advantages and they need to be supplemented with additional staining for elastic component.

Various histological techniques have been evolved for the demonstration of elastic fibers. Verhoeff’s iron hematoxylin was commonly used in combination with other polychrome methods (Buk 1984; Garvey 1984; Garvey et al. 1986, 1987; O’Connor and Valle 1982), but it has several disadvantages. It requires a very precise differentiation in ferric chloride for each batch of tissue sections, and hence, it cannot be freely used in automatic stainers. Furthermore, it does not demonstrate the finest elastic fibers and gives poor color separation in combination with polychrome methods. Weigert’s resorcin–fuchsin method requires more steps and needs boiling of the solution, and result of staining highly depends of the batch of basic fuchsin used. We found synthetic orcein to be superior in staining of elastica. Unlike Verhoeff’s method, it is a progressive stain and may require no further differentiation. Since it is simple to use (only one dye solution) and gives selective staining of elastic components, orcein has been preferred in a number of studies (listed in Henwood 2003). We have successfully used orcein alone in our previous study (Jawien et al. 2006).

So far, only few reports have presented combinations of elastic fiber staining with multicolor staining of the connective tissue. Orcein was used in combination with picroindigocarmine (Steven et al. 2000; Demirtas et al. 2007). Although this method satisfactorily demonstrates components of the connective tissue, cartilage, and bone, it does not offer sufficient contrast for automatic detection of plaque and vessel components. O’Connor and Valle (1982) combined Verhoeff’s hematoxylin with Masson’s trichrome, but this technique failed to differentiate fibrin.

Polychrome stainings based on application of dyes with various molecular weights allow discrimination of early, mature, and old fibrin, as was discussed for the case of MSB by Lendrum et al. (1962). MSB together with routine HE and Pearls’ prussian blue stainings on separate sections were suggested to be preferably used for the detection of intraplaque hemorrhages in apoE^−/−^ mice to achieve maximal reliability (Sluimer et al. 2015). MSB was also combined with elastic tissue stain by Buk (1984). A similar method was presented by Garvey et al. (1987), although they applied, alternatively, other dyestuff to detect fibrin (lysamine fast yellow, Biebrich scarlet, acid fuchsin, and ponceau 2R).

Some studies assessing atherosclerosis employed Movat pentachrome to stain plaque components (e.g., Carr et al. 1996). In Garvey et al. (1986) and Silverman (1972) modifications of this method, iron hematoxylin and orcein were used to contrast elastic fibers. Movat pentachrome very well demonstrates collagen, elastic fibers, and mature fibrin, and gives good contrast with erythrocytes. Unlike other polychrome methods, it also stains mucus, as well as proteoglycans and glycoproteins of the extracellular matrix. However, Movat pentachrome is a complex technique, not suited for automatic staining. It requires highly toxic mercuric chloride (sublimate) for fixation or post-fixation steps and utilizes very expensive Spanish saffron. Comparison of all cited polychrome stainings and method presented in the current study is summarized in Table 1.

**Table 1:** Comparison of polychrome stainings combined with stainings for elastic components

Staining method	Nuclei	Cytoplasm	Erythrocytes	Muscle	Elastic fibers	Collagen fibers	Early fibrin	Mature fibrin	Old fibrin	Muco-substances	Time (h)	Automatic stainers	References
Verhoeff + Masson trichrome	Blue/black	Red	Red	Red	Black	Green or blue	–	Red	–	–	2	No	O’Connor and Valle (1982)
Verhoeff + modified Masson	Blue/black	Red	Red	Red	Blue/black	Green or blue	–	Red	–	–	1	No	Garvey (1984)
Verhoeff + MSB	Blue/black	Red	Yellow	Red	Blue/black	Blue	Yellow	Red	Blue	–	1.5	No	Buk (1984)
Verhoeff + modified MSB	Black	Red	Yellow	Red	Black	Green or blue	Yellow	Red	Blue	–	1	No	Garvey et al. (1987)
Iron hematoxylin + MSB	Black	Red	Yellow	Red	Light blue	Blue	Yellow	Red	Blue	–	1.5	Yes	Sluimer et al. (2015)
Verhoeff + movat pentachrome	Black	Red	Red	Red	Black	Yellow	–	Red	–	Turquois	1.5	No	Garvey et al. (1986)
Verhoeff + orcein+movat pentachrome	Black	Red	Red	Red	Purple to black	Yellow	–	Red	–	Turquois	1.5	No	Silverman (1972)
Orcein + picroindigocarmine*	Rust to dark brown	–	Yellowish green	Yellowish brown to yellow green	Brown	Greenish blue	–	–	–	–	1	Yes	Steven et al. (2000) Demirtas et al. (1962)
Orcein (synthetic) + MSB (no hematoxylin)	Red	Red	Yellow	Red	Purple	Blue	Yellow	Red	Blue	–	>1	Yes	This study

*Other components are stained as follows: cartilage matrix—pink, chondroblasts—blue, and bone matrix—dark blue

Polychrome methods allow simultaneous demonstration of various tissue components. Obviously, similar results can be achieved by applying individual specific stainings to consecutive tissue sections (e.g., sirius red for collagen, immunohistochemistry for fibrin, and glycophorin immunohistochemistry for erythrocytes) and using separate slides for staining of elastic components. However, the number of sections available from the brachiocephalic trunk is limited because of its small size (approximately 1 mm). Since distribution of atherosclerotic plaques differs individually, numerous sections from the entire BCA should be studied with increment not exceeding 50 μm. The use of polychrome methods allows to save sections for additional stainings that cannot be combined with polychrome techniques, e.g., alizarin red for calcium deposits, Pearls prussian blue for iron, alcian blue for proteoglycans, or immunohistochemistry for endothelial cells, macrophages, lymphocytes, smooth muscle cells, and matrix proteins.

Recently, we have successfully used OMSB staining for qualitative analysis of BCAs in apoE/LDLR^−/−^ mice fed pro-atherogenic low carbohydrate high protein diet (Kostogrys et al. 2015). Since OMSB provides good contrast and sufficient color separation for automatic segmentation, we developed a method for computer-assisted image analysis that allowed to recognize vessel media, lumen and entire plaque, and its components—lipids and areas rich with collagen fibers. Such data were further processed providing information about crucial morphometric parameters of atherosclerotic artery, including vessel obliteration, plaque size, and plaque type. In this study, we presented an example of quantitative assessment of BCA plaques using our approach. It demonstrated clear-cut differences in atherosclerotic plaque distribution in proximal and distal segments of BCA. We clearly showed that distal part of BCA displayed larger total vessel area, vessel media, and lumen, as well as plaques, with higher areas occupied by lipids and collagen fibers. Larger media, total vessel area, and lumen in distal BCA may reflect the morphology of the artery before its branching into internal and external carotid arteries. Larger absolute and relative plaque areas, as well as absolute areas occupied by lipids and collagen may be indicative for more advanced lesions observed in distal portion of BCA. The results support the value of OMSB staining for use in automatic quantitative analysis of tissue components of atherosclerotic plaques.

In conclusion, combined orcein and MSB staining provides reliable color contrast to distinguish numerous constituents of atherosclerotic plaques including determinants of plaque instability. The method is simple and economic, and can be adapted to automatic stainers. Therefore, we recommend OMSB staining for routine use not only in qualitative but also in quantitative microscopic assessment of atherosclerotic lesions in formalin-fixed and paraffin-embedded arteries.
